# Reduced myocardial perfusion in post renal transplant population is not associated with aortic stiffness

**DOI:** 10.1186/1532-429X-16-S1-P226

**Published:** 2014-01-16

**Authors:** Susie F Parnham, Suchi Grover, Craig Bradbrook, Darryl Leong, Carmine De Pasquale, Jonathan Gleadle, Joseph Selvanayagam

**Affiliations:** 1 Cardiology, Flinders University, Flinders Medical Centre, Bedford Park, South Australia, Australia; 2 Renal Medicine, Flinders Medical Centre, Bedford Park, South Australia, Australia; 3 Flinders Centre for Cardiovascular Magnetic Resonance Research, Flinders Medical Centre, Bedford Park, South Australia, Australia; 4 School of Medicine, Flinders University, Bedford Park, South Australia, Australia

## Background

Objective: The purpose of this study was to assess central (aortic) vascular dysfunction in post renal transplant patients by high-resolution cardiovascular magnetic resonance imaging (CMR). Background: Renal transplant recipients are at increased risk of cardiovascular (CV) disease. The cardiac phenotype in post-transplant recipients is not well defined. A recent study suggested myocardial perfusion is impaired in renal transplant patients irrespective of the degree of left ventricular hypertrophy[1].We hypothesized that post transplant patients have persistently increased aortic stiffness, and that it could be correlated with reduced myocardial perfusion.

## Methods

Methods: Twenty-five asymptomatic renal transplant patients (RT) and 17 hypertensive controls (HT) underwent CMR scanning at 1.5 T. Myocardial function, late enhancement, aortic pulse wave velocity and first-pass perfusion at rest and stress was performed. Myocardial Perfusion Reserve Index (MPRI) was calculated as the ratio of hyperemic to resting myocardial blood flow by dividing the myocardial perfusion at stress by rest perfusion (corrected to rate pressure product). Pulse wave velocity (PWV) was calculated as the ratio between the distance and time from ascending aorta to descending aorta at the level of main pulmonary artery. Statistical analyses were performed using mixed effects modelling for MPRI and linear regression for relationship between MPRI and PWV.

## Results

Results: Baseline clinical characteristics were similar for RT and HT control groups. Mean inter-ventricular septal thickness and LV mass indexed to body surface area were similar in both RT cases and HT controls. MPRI was significantly lower in the RT group compared to the HT group (1.36 ± 0.52 vs 2.06 ± 0.59, p = 0.0004), globally and in all three coronary artery territories[1]. Aortic Pulse Wave Velocity (PWV) is not significantly different between RT and HT group (3.46 ± 0.51 vs 5.21 ± 1.07, p = 0.14). There is no correlation between aortic pulse wave velocity and MPRI (r2 = 0.0026, coeff = 0.18, p = 0.83).

## Conclusions

Conclusions: In our cohort population, there is no evidence of increased aortic stiffness in post renal transplant patients compared to hypertensive patients. Although these patients demonstrate impaired myocardial perfusion reserve, this is not correlated with increased aortic stiffness.

## Funding

None.
